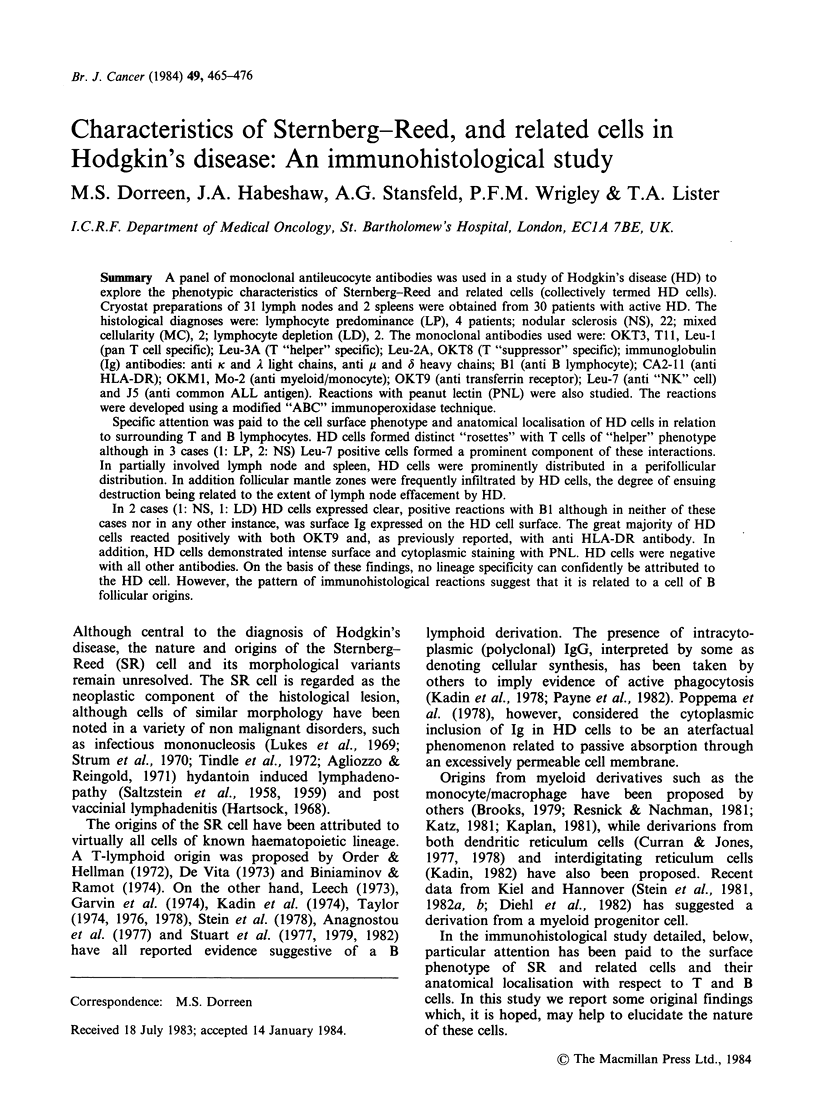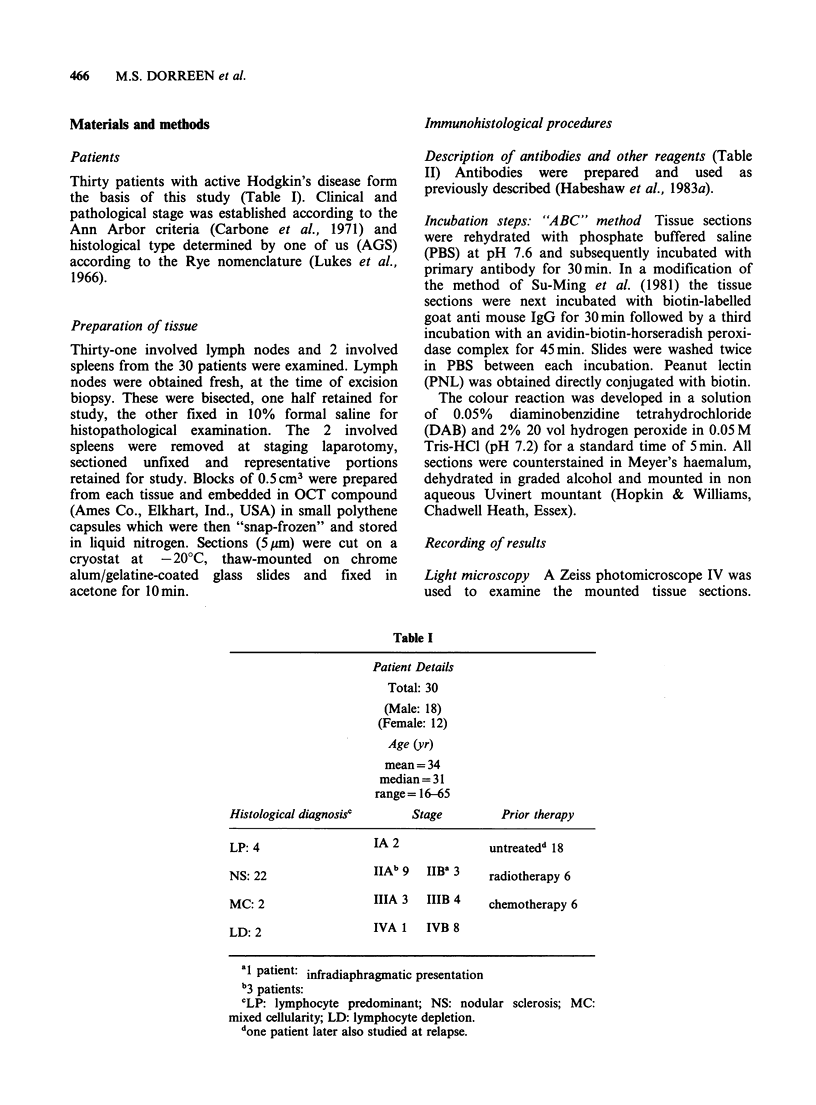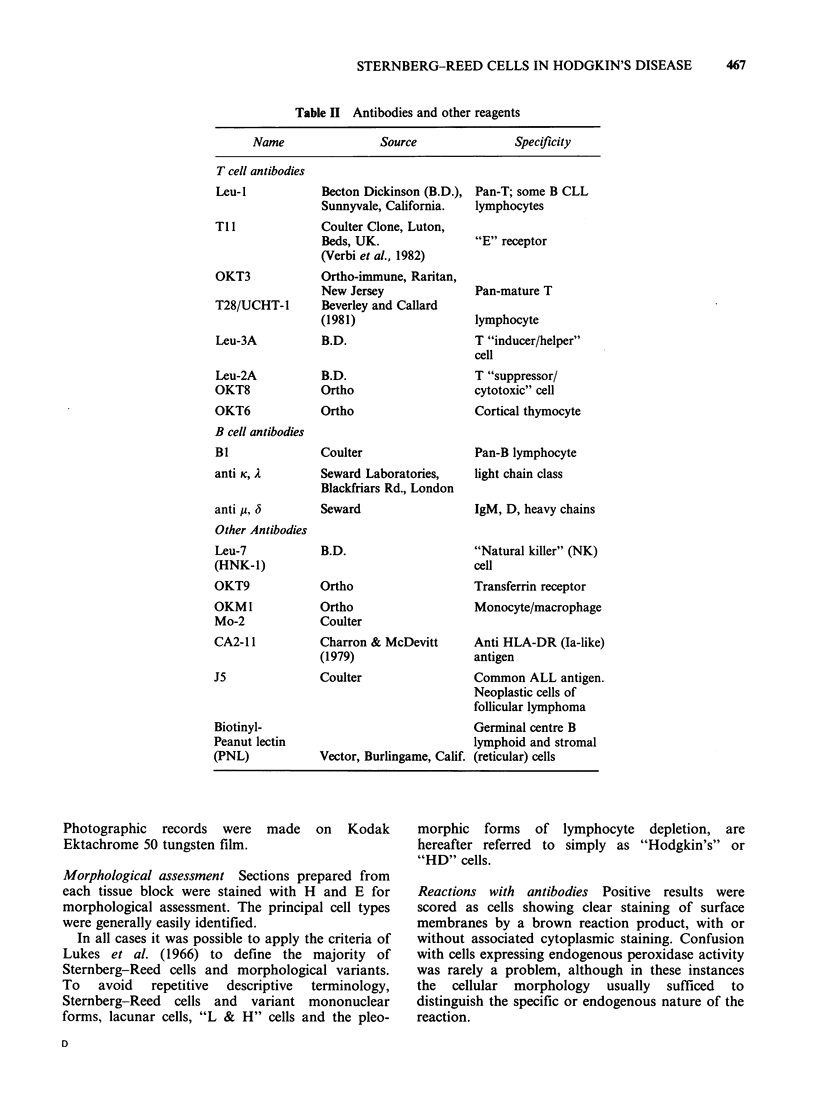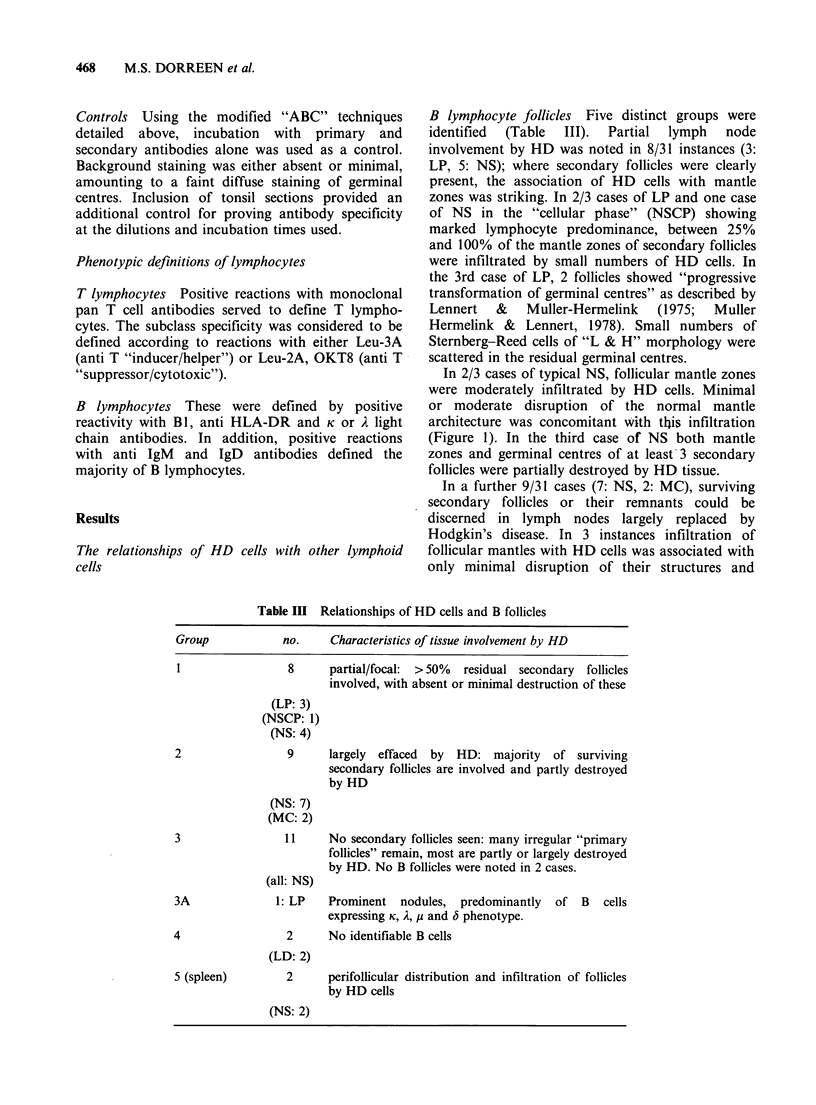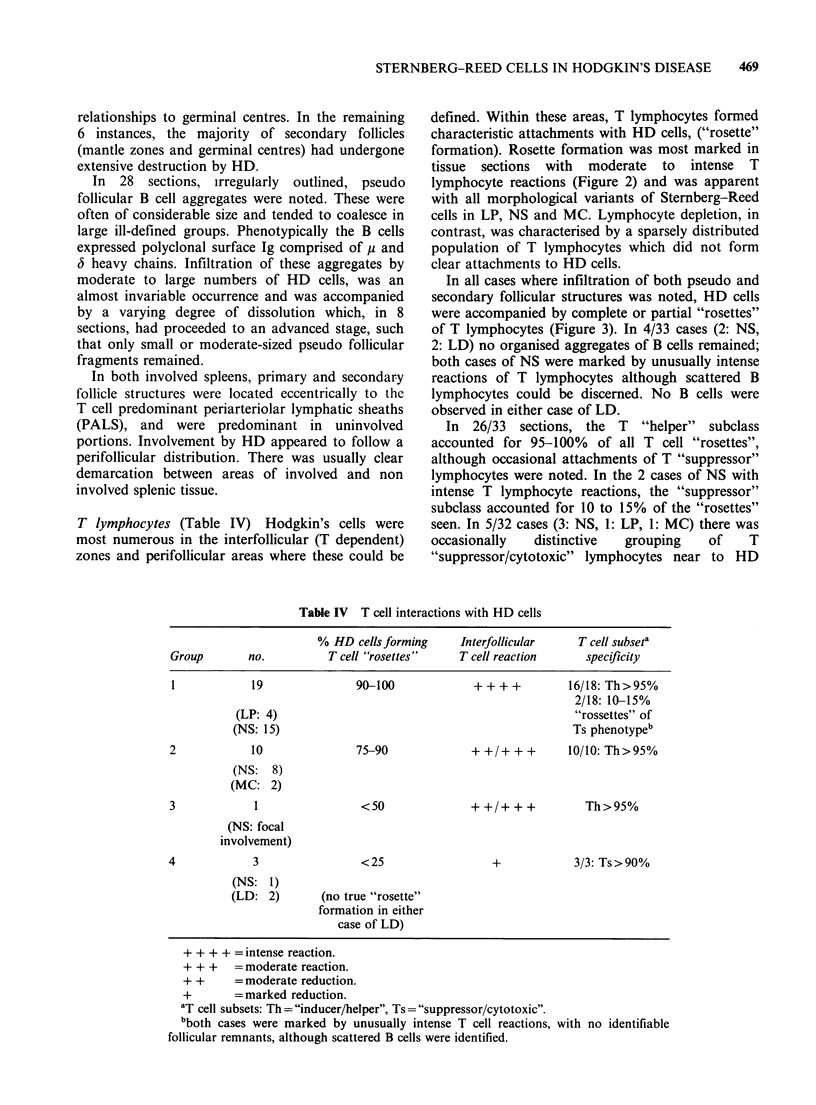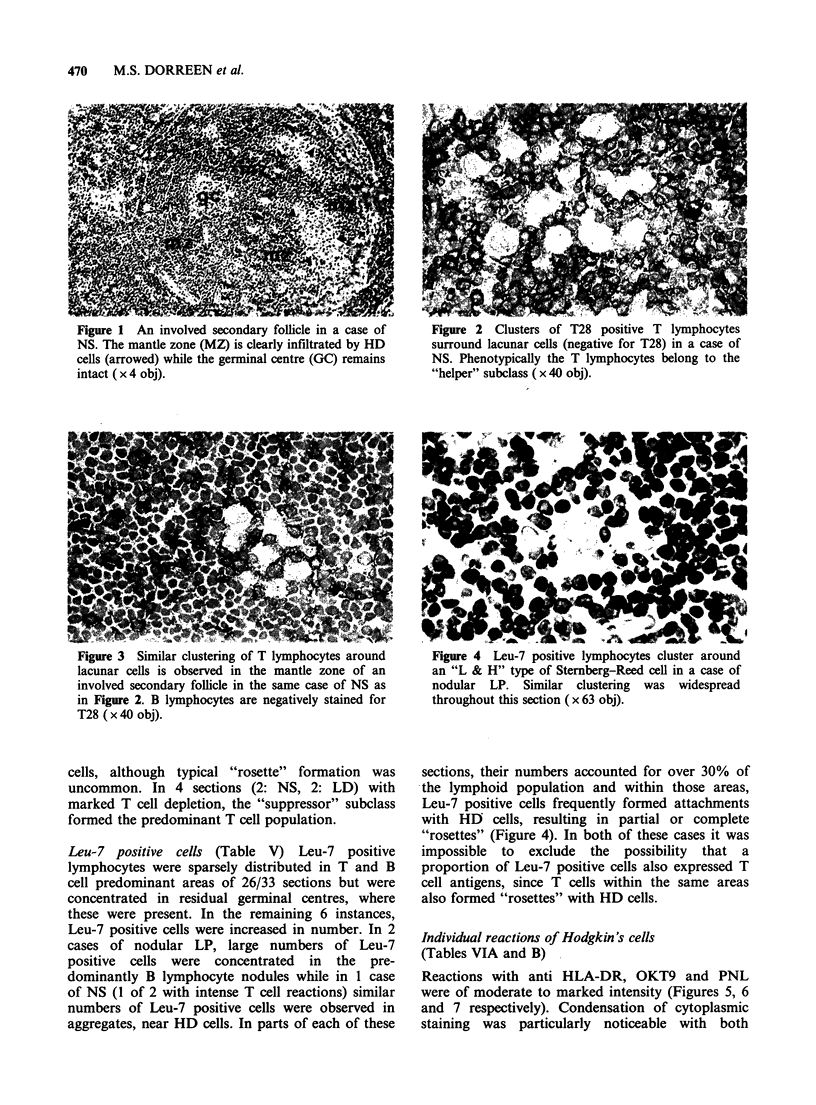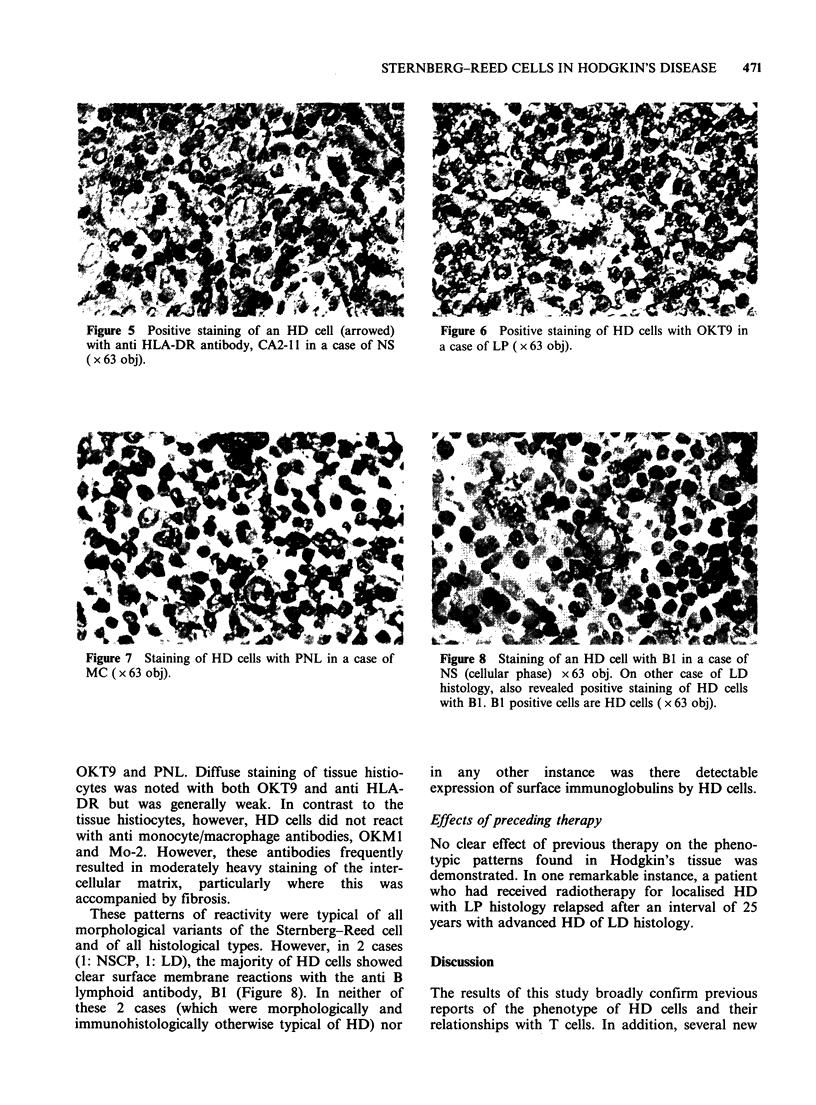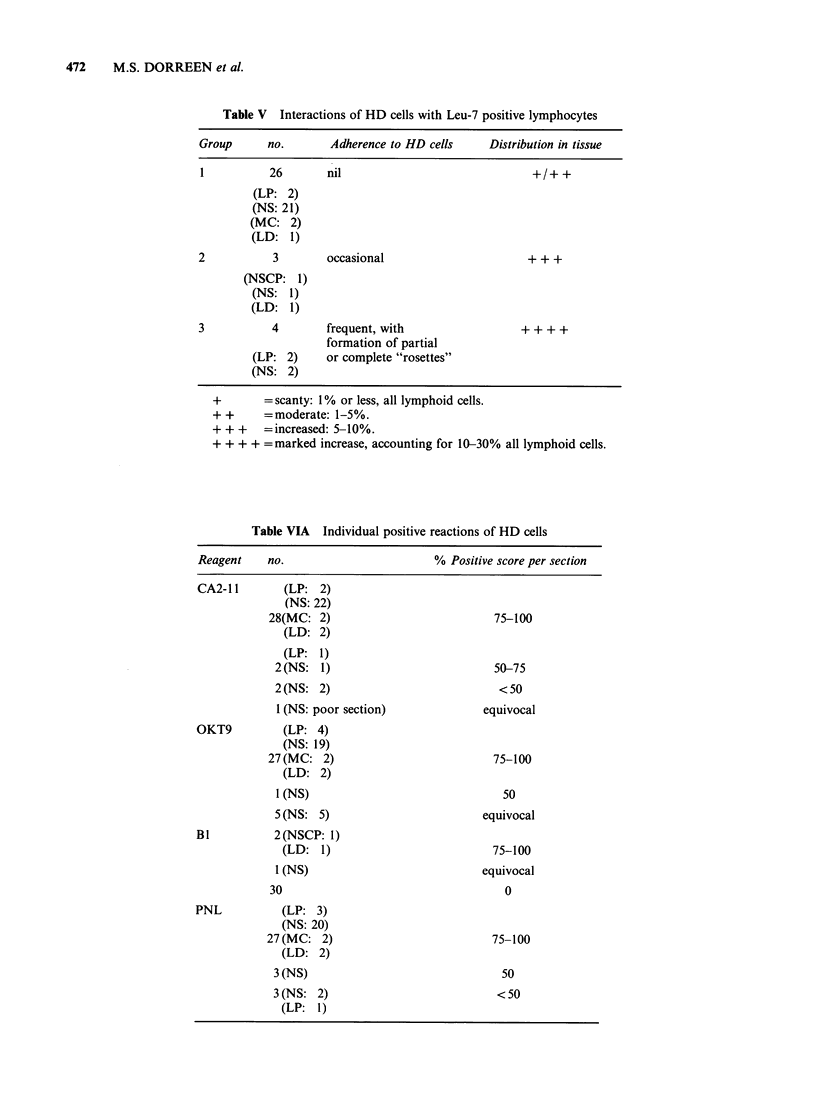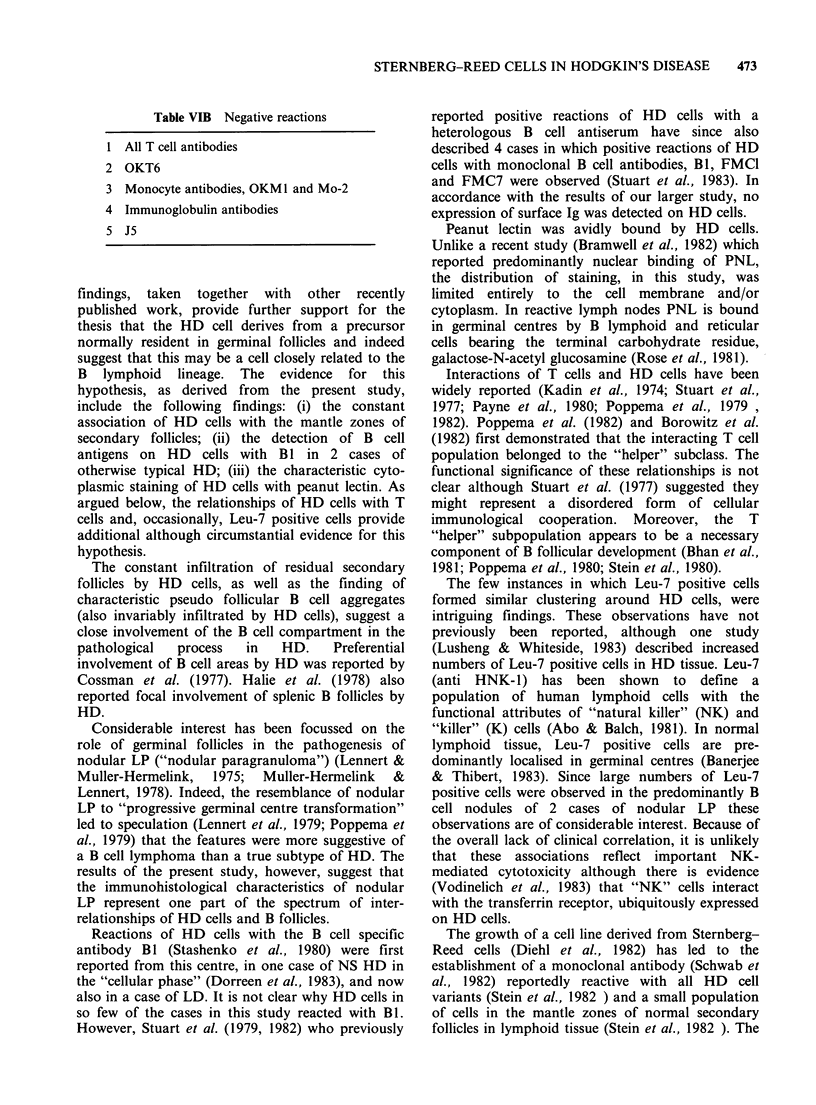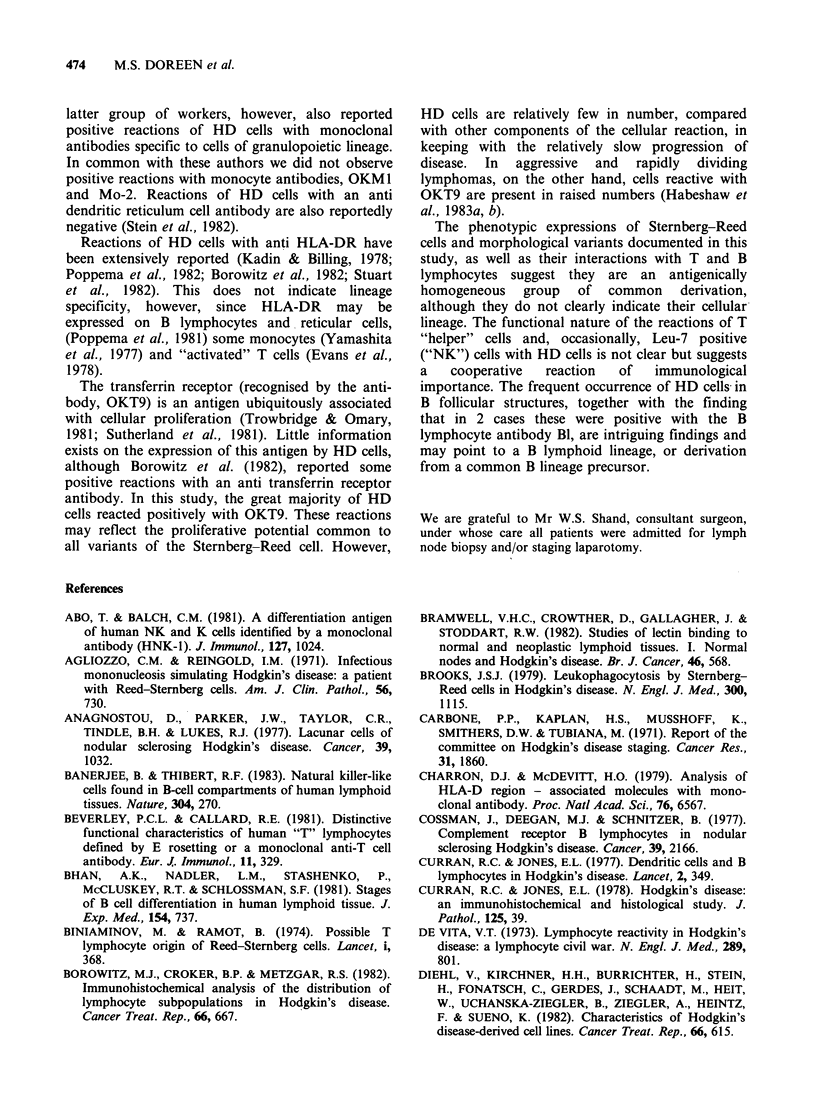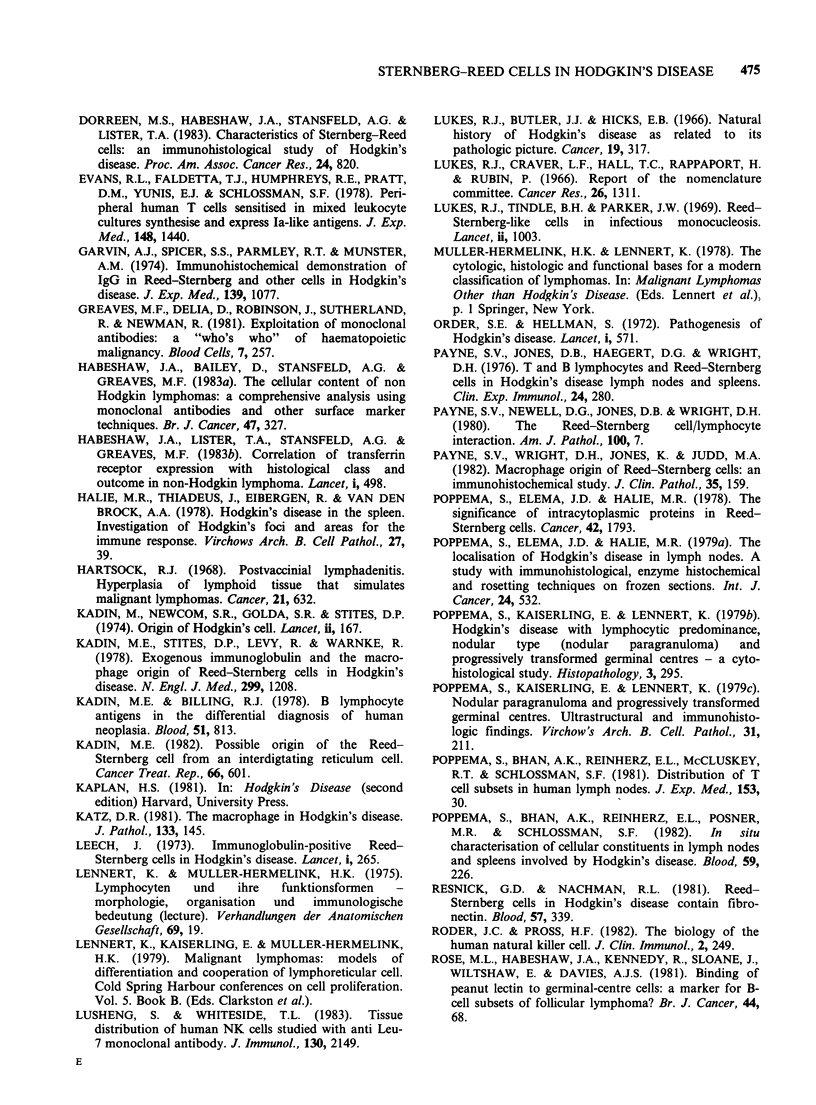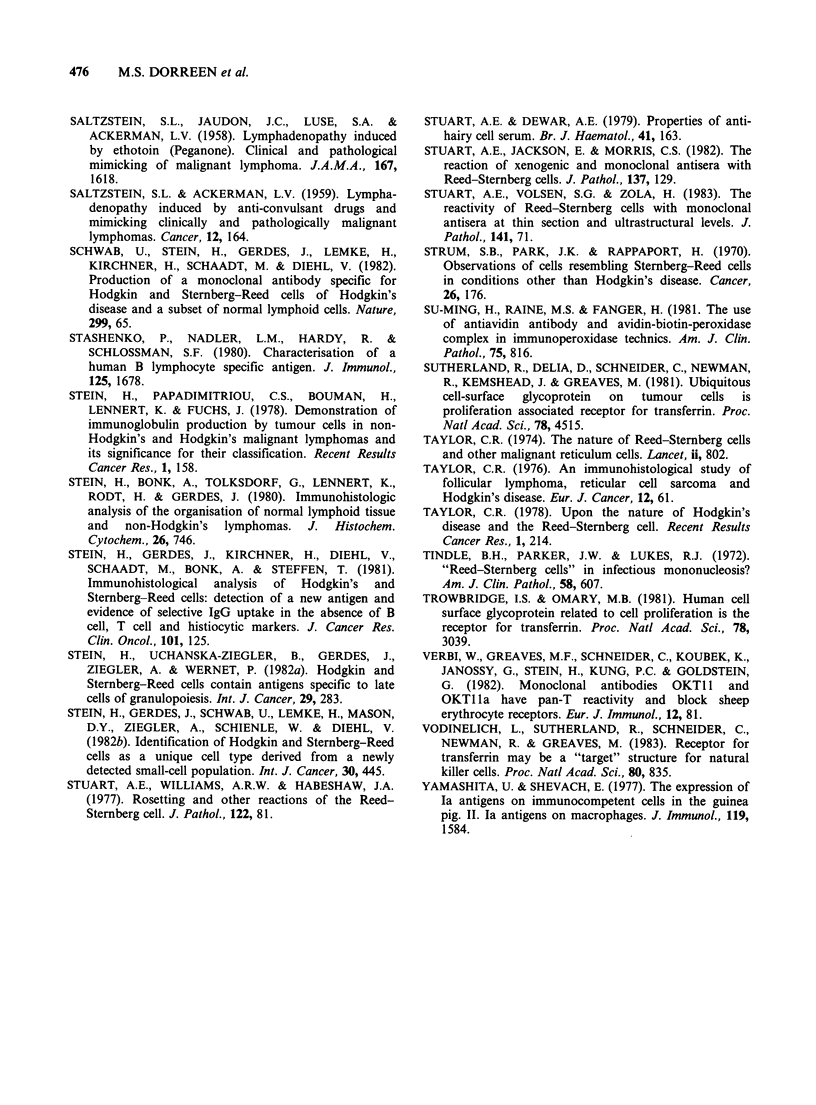# Characteristics of Sternberg-Reed, and related cells in Hodgkin's disease: an immunohistological study.

**DOI:** 10.1038/bjc.1984.74

**Published:** 1984-04

**Authors:** M. S. Dorreen, J. A. Habeshaw, A. G. Stansfeld, P. F. Wrigley, T. A. Lister

## Abstract

**Images:**


*Br. J. Cancer* (1984) **49**, 465–476

### Characteristics of Sternberg–Reed, and related cells in Hodgkin's disease: An immunohistological study

M.S. Dorreen, J.A. Habeshaw, A.G. Stansfeld, P.F.M. Wrigley & T.A. Lister

*I.C.R.F. Department of Medical Oncology, St. Bartholomew's Hospital, London, EC1A 7BE, UK.*

**Summary** A panel of monoclonal antileucocyte antibodies was used in a study of Hodgkin's disease (HD) to explore the phenotypic characteristics of Sternberg–Reed and related cells (collectively termed HD cells). Cryostat preparations of 31 lymph nodes and 2 spleens were obtained from 30 patients with active HD. The histological diagnoses were: lymphocyte predominance (LP), 4 patients; nodular sclerosis (NS), 22; mixed cellularity (MC), 2; lymphocyte depletion (LD), 2. The monoclonal antibodies used were: OKT3, T11, Leu-1 (pan T cell specific); Leu-3A (T "helper" specific); Leu-2A, OKT8 (T "suppressor" specific); immunoglobulin (Ig) antibodies: anti $\kappa$ and $\lambda$ light chains, anti $\mu$ and $\delta$ heavy chains; B1 (anti B lymphocyte); CA2-11 (anti HLA-DR); OKM1, Mo-2 (anti myeloid/monocyte); OKT9 (anti transferrin receptor); Leu-7 (anti "NK" cell) and J5 (anti common ALL antigen). Reactions with peanut lectin (PNL) were also studied. The reactions were developed using a modified "ABC" immunoperoxidase technique.

Specific attention was paid to the cell surface phenotype and anatomical localisation of HD cells in relation to surrounding T and B lymphocytes. HD cells formed distinct "rosettes" with T cells of "helper" phenotype although in 3 cases (1: LP, 2: NS) Leu-7 positive cells formed a prominent component of these interactions. In partially involved lymph node and spleen, HD cells were prominently distributed in a perifollicular distribution. In addition follicular mantle zones were frequently infiltrated by HD cells, the degree of ensuing destruction being related to the extent of lymph node effacement by HD.

In 2 cases (1: NS, 1: LD) HD cells expressed clear, positive reactions with B1 although in neither of these cases nor in any other instance, was surface Ig expressed on the HD cell surface. The great majority of HD cells reacted positively with both OKT9 and, as previously reported, with anti HLA-DR antibody. In addition, HD cells demonstrated intense surface and cytoplasmic staining with PNL. HD cells were negative with all other antibodies. On the basis of these findings, no lineage specificity can confidently be attributed to the HD cell. However, the pattern of immunohistological reactions suggest that it is related to a cell of B follicular origins.

Although central to the diagnosis of Hodgkin's disease, the nature and origins of the Sternberg–Reed (SR) cell and its morphological variants remain unresolved. The SR cell is regarded as the neoplastic component of the histological lesion, although cells of similar morphology have been noted in a variety of non malignant disorders, such as infectious mononucleosis (Lukes *et al.*, 1969; Strum *et al.*, 1970; Tindle *et al.*, 1972; Agliozzo & Reingold, 1971) hydantoin induced lymphadenopathy (Saltzstein *et al.*, 1958, 1959) and post vaccinial lymphadenitis (Hartsock, 1968).

The origins of the SR cell have been attributed to virtually all cells of known haematopoietic lineage. A T-lymphoid origin was proposed by Order & Hellman (1972), De Vita (1973) and Biniaminov & Ramot (1974). On the other hand, Leech (1973), Garvin *et al.* (1974), Kadin *et al.* (1974), Taylor (1974, 1976, 1978), Stein *et al.* (1978), Anagnostou *et al.* (1977) and Stuart *et al.* (1977, 1979, 1982) have all reported evidence suggestive of a B lymphoid derivation. The presence of intracytoplasmic (polyclonal) IgG, interpreted by some as denoting cellular synthesis, has been taken by others to imply evidence of active phagocytosis (Kadin *et al.*, 1978; Payne *et al.*, 1982). Poppema *et al.* (1978), however, considered the cytoplasmic inclusion of Ig in HD cells to be an aterfactual phenomenon related to passive absorption through an excessively permeable cell membrane.

Origins from myeloid derivatives such as the monocyte/macrophage have been proposed by others (Brooks, 1979; Resnick & Nachman, 1981; Katz, 1981; Kaplan, 1981), while derivarions from both dendritic reticulum cells (Curran & Jones, 1977, 1978) and interdigitating reticulum cells (Kadin, 1982) have also been proposed. Recent data from Kiel and Hannover (Stein *et al.*, 1981, 1982*a*, *b*; Diehl *et al.*, 1982) has suggested a derivation from a myeloid progenitor cell.

In the immunohistological study detailed, below, particular attention has been paid to the surface phenotype of SR and related cells and their anatomical localisation with respect to T and B cells. In this study we report some original findings which, it is hoped, may help to elucidate the nature of these cells.

Correspondence: M.S. Dorreen

Received 18 July 1983; accepted 14 January 1984.

© The Macmillan Press Ltd., 1984

### Materials and methods

### *Patients*

Thirty patients with active Hodgkin's disease form the basis of this study (Table I). Clinical and pathological stage was established according to the Ann Arbor criteria (Carbone *et al.*, 1971) and histological type determined by one of us (AGS) according to the Rye nomenclature (Lukes *et al.*, 1966).

### *Preparation of tissue*

Thirty-one involved lymph nodes and 2 involved spleens from the 30 patients were examined. Lymph nodes were obtained fresh, at the time of excision biopsy. These were bisected, one half retained for study, the other fixed in 10% formal saline for histopathological examination. The 2 involved spleens were removed at staging laparotomy, sectioned unfixed and representative portions retained for study. Blocks of 0.5 $cm^3$ were prepared from each tissue and embedded in OCT compound (Ames Co., Elkhart, Ind., USA) in small polythene capsules which were then "snap-frozen" and stored in liquid nitrogen. Sections (5 $\mu$m) were cut on a cryostat at $-20°$C, thaw-mounted on chrome alum/gelatine-coated glass slides and fixed in acetone for 10 min.

### *Immunohistological procedures*

*Description of antibodies and other reagents* (Table II) Antibodies were prepared and used as previously described (Habeshaw *et al.*, 1983*a*).

*Incubation steps: "ABC" method* Tissue sections were rehydrated with phosphate buffered saline (PBS) at pH 7.6 and subsequently incubated with primary antibody for 30 min. In a modification of the method of Su-Ming *et al.* (1981) the tissue sections were next incubated with biotin-labelled goat anti mouse IgG for 30 min followed by a third incubation with an avidin-biotin-horseradish peroxidase complex for 45 min. Slides were washed twice in PBS between each incubation. Peanut lectin (PNL) was obtained directly conjugated with biotin.

The colour reaction was developed in a solution of 0.05% diaminobenzidine tetrahydrochloride (DAB) and 2% 20 vol hydrogen peroxide in 0.05 M Tris-HCl (pH 7.2) for a standard time of 5 min. All sections were counterstained in Meyer's haemalum, dehydrated in graded alcohol and mounted in non aqueous Uvinert mountant (Hopkin & Williams, Chadwell Heath, Essex).

### *Recording of results*

*Light microscopy* A Zeiss photomicroscope IV was used to examine the mounted tissue sections.

**Table I**

*Patient Details*

Total: 30

(Male: 18)
(Female: 12)

*Age (yr)*

mean = 34
median = 31
range = 16–65

| *Histological diagnosis*[c] | *Stage* | | *Prior therapy* |
|---|---|---|---|
| LP: 4 | IA 2 | | untreated[d] 18 |
| NS: 22 | IIA[b] 9 | IIB[a] 3 | radiotherapy 6 |
| MC: 2 | IIIA 3 | IIIB 4 | chemotherapy 6 |
| LD: 2 | IVA 1 | IVB 8 | |

[a]1 patient: infradiaphragmatic presentation
[b]3 patients:
[c]LP: lymphocyte predominant; NS: nodular sclerosis; MC: mixed cellularity; LD: lymphocyte depletion.
[d]one patient later also studied at relapse.

**Table II** Antibodies and other reagents

| *Name* | *Source* | *Specificity* |
|---|---|---|
| *T cell antibodies* | | |
| Leu-1 | Becton Dickinson (B.D.), Sunnyvale, California. | Pan-T; some B CLL lymphocytes |
| T11 | Coulter Clone, Luton, Beds, UK. (Verbi *et al.*, 1982) | "E" receptor |
| OKT3 | Ortho-immune, Raritan, New Jersey | Pan-mature T |
| T28/UCHT-1 | Beverley and Callard (1981) | lymphocyte |
| Leu-3A | B.D. | T "inducer/helper" cell |
| Leu-2A | B.D. | T "suppressor/ |
| OKT8 | Ortho | cytotoxic" cell |
| OKT6 | Ortho | Cortical thymocyte |
| *B cell antibodies* | | |
| B1 | Coulter | Pan-B lymphocyte |
| anti $\kappa$, $\lambda$ | Seward Laboratories, Blackfriars Rd., London | light chain class |
| anti $\mu$, $\delta$ | Seward | IgM, D, heavy chains |
| *Other Antibodies* | | |
| Leu-7 (HNK-1) | B.D. | "Natural killer" (NK) cell |
| OKT9 | Ortho | Transferrin receptor |
| OKM1 | Ortho | Monocyte/macrophage |
| Mo-2 | Coulter | |
| CA2-11 | Charron & McDevitt (1979) | Anti HLA-DR (Ia-like) antigen |
| J5 | Coulter | Common ALL antigen. Neoplastic cells of follicular lymphoma |
| Biotinyl-Peanut lectin (PNL) | Vector, Burlingame, Calif. | Germinal centre B lymphoid and stromal (reticular) cells |

Photographic records were made on Kodak Ektachrome 50 tungsten film.

*Morphological assessment* Sections prepared from each tissue block were stained with H and E for morphological assessment. The principal cell types were generally easily identified.

In all cases it was possible to apply the criteria of Lukes *et al.* (1966) to define the majority of Sternberg–Reed cells and morphological variants. To avoid repetitive descriptive terminology, Sternberg–Reed cells and variant mononuclear forms, lacunar cells, "L & H" cells and the pleomorphic forms of lymphocyte depletion, are hereafter referred to simply as "Hodgkin's" or "HD" cells.

*Reactions with antibodies* Positive results were scored as cells showing clear staining of surface membranes by a brown reaction product, with or without associated cytoplasmic staining. Confusion with cells expressing endogenous peroxidase activity was rarely a problem, although in these instances the cellular morphology usually sufficed to distinguish the specific or endogenous nature of the reaction.

*Controls* Using the modified "ABC" techniques detailed above, incubation with primary and secondary antibodies alone was used as a control. Background staining was either absent or minimal, amounting to a faint diffuse staining of germinal centres. Inclusion of tonsil sections provided an additional control for proving antibody specificity at the dilutions and incubation times used.

*Phenotypic definitions of lymphocytes*

*T lymphocytes* Positive reactions with monoclonal pan T cell antibodies served to define T lymphocytes. The subclass specificity was considered to be defined according to reactions with either Leu-3A (anti T "inducer/helper") or Leu-2A, OKT8 (anti T "suppressor/cytotoxic").

*B lymphocytes* These were defined by positive reactivity with B1, anti HLA-DR and $\kappa$ or $\lambda$ light chain antibodies. In addition, positive reactions with anti IgM and IgD antibodies defined the majority of B lymphocytes.

### Results

*The relationships of HD cells with other lymphoid cells*

*B lymphocyte follicles* Five distinct groups were identified (Table III). Partial lymph node involvement by HD was noted in 8/31 instances (3: LP, 5: NS); where secondary follicles were clearly present, the association of HD cells with mantle zones was striking. In 2/3 cases of LP and one case of NS in the "cellular phase" (NSCP) showing marked lymphocyte predominance, between 25% and 100% of the mantle zones of secondary follicles were infiltrated by small numbers of HD cells. In the 3rd case of LP, 2 follicles showed "progressive transformation of germinal centres" as described by Lennert & Muller-Hermelink (1975; Muller Hermelink & Lennert, 1978). Small numbers of Sternberg–Reed cells of "L & H" morphology were scattered in the residual germinal centres.

In 2/3 cases of typical NS, follicular mantle zones were moderately infiltrated by HD cells. Minimal or moderate disruption of the normal mantle architecture was concomitant with this infiltration (Figure 1). In the third case of NS both mantle zones and germinal centres of at least 3 secondary follicles were partially destroyed by HD tissue.

In a further 9/31 cases (7: NS, 2: MC), surviving secondary follicles or their remnants could be discerned in lymph nodes largely replaced by Hodgkin's disease. In 3 instances infiltration of follicular mantles with HD cells was associated with only minimal disruption of their structures and

**Table III** Relationships of HD cells and B follicles

| *Group* | *no.* | *Characteristics of tissue involvement by HD* |
|---|---|---|
| 1 | 8<br>(LP: 3)<br>(NSCP: 1)<br>(NS: 4) | partial/focal: >50% residual secondary follicles involved, with absent or minimal destruction of these |
| 2 | 9<br>(NS: 7)<br>(MC: 2) | largely effaced by HD: majority of surviving secondary follicles are involved and partly destroyed by HD |
| 3 | 11<br>(all: NS) | No secondary follicles seen: many irregular "primary follicles" remain, most are partly or largely destroyed by HD. No B follicles were noted in 2 cases. |
| 3A | 1: LP | Prominent nodules, predominantly of B cells expressing $\kappa$, $\lambda$, $\mu$ and $\delta$ phenotype. |
| 4 | 2<br>(LD: 2) | No identifiable B cells |
| 5 (spleen) | 2<br>(NS: 2) | perifollicular distribution and infiltration of follicles by HD cells |

relationships to germinal centres. In the remaining 6 instances, the majority of secondary follicles (mantle zones and germinal centres) had undergone extensive destruction by HD.

In 28 sections, irregularly outlined, pseudo follicular B cell aggregates were noted. These were often of considerable size and tended to coalesce in large ill-defined groups. Phenotypically the B cells expressed polyclonal surface Ig comprised of $\mu$ and $\delta$ heavy chains. Infiltration of these aggregates by moderate to large numbers of HD cells, was an almost invariable occurrence and was accompanied by a varying degree of dissolution which, in 8 sections, had proceeded to an advanced stage, such that only small or moderate-sized pseudo follicular fragments remained.

In both involved spleens, primary and secondary follicle structures were located eccentrically to the T cell predominant periarteriolar lymphatic sheaths (PALS), and were predominant in uninvolved portions. Involvement by HD appeared to follow a perifollicular distribution. There was usually clear demarcation between areas of involved and non involved splenic tissue.

*T lymphocytes* (Table IV) Hodgkin's cells were most numerous in the interfollicular (T dependent) zones and perifollicular areas where these could be defined. Within these areas, T lymphocytes formed characteristic attachments with HD cells, ("rosette" formation). Rosette formation was most marked in tissue sections with moderate to intense T lymphocyte reactions (Figure 2) and was apparent with all morphological variants of Sternberg–Reed cells in LP, NS and MC. Lymphocyte depletion, in contrast, was characterised by a sparsely distributed population of T lymphocytes which did not form clear attachments to HD cells.

In all cases where infiltration of both pseudo and secondary follicular structures was noted, HD cells were accompanied by complete or partial "rosettes" of T lymphocytes (Figure 3). In 4/33 cases (2: NS, 2: LD) no organised aggregates of B cells remained; both cases of NS were marked by unusually intense reactions of T lymphocytes although scattered B lymphocytes could be discerned. No B cells were observed in either case of LD.

In 26/33 sections, the T "helper" subclass accounted for 95–100% of all T cell "rosettes", although occasional attachments of T "suppressor" lymphocytes were noted. In the 2 cases of NS with intense T lymphocyte reactions, the "suppressor" subclass accounted for 10 to 15% of the "rosettes" seen. In 5/32 cases (3: NS, 1: LP, 1: MC) there was occasionally distinctive grouping of T "suppressor/cytotoxic" lymphocytes near to HD

**Table IV** T cell interactions with HD cells

| *Group* | *no.* | *% HD cells forming T cell "rosettes"* | *Interfollicular T cell reaction* | *T cell subset*[a] *specificity* |
|---|---|---|---|---|
| 1 | 19<br>(LP: 4)<br>(NS: 15) | 90–100 | + + + + | 16/18: Th > 95%<br>2/18: 10–15% "rossettes" of Ts phenotype[b] |
| 2 | 10<br>(NS: 8)<br>(MC: 2) | 75–90 | + +/+ + + | 10/10: Th > 95% |
| 3 | 1<br>(NS: focal involvement) | < 50 | + +/+ + + | Th > 95% |
| 4 | 3<br>(NS: 1)<br>(LD: 2) | < 25<br>(no true "rosette" formation in either case of LD) | + | 3/3: Ts > 90% |

+ + + + = intense reaction.
+ + + = moderate reaction.
+ + = moderate reduction.
+ = marked reduction.
[a]T cell subsets: Th = "inducer/helper", Ts = "suppressor/cytotoxic".
[b]both cases were marked by unusually intense T cell reactions, with no identifiable follicular remnants, although scattered B cells were identified.

**Figure 1** An involved secondary follicle in a case of NS. The mantle zone (MZ) is clearly infiltrated by HD cells (arrowed) while the germinal centre (GC) remains intact (×4 obj).

**Figure 2** Clusters of T28 positive T lymphocytes surround lacunar cells (negative for T28) in a case of NS. Phenotypically the T lymphocytes belong to the "helper" subclass (×40 obj).

**Figure 3** Similar clustering of T lymphocytes around lacunar cells is observed in the mantle zone of an involved secondary follicle in the same case of NS as in **Figure 2**. B lymphocytes are negatively stained for T28 (×40 obj).

**Figure 4** Leu-7 positive lymphocytes cluster around an "L & H" type of Sternberg–Reed cell in a case of nodular LP. Similar clustering was widespread throughout this section (×63 obj).

cells, although typical "rosette" formation was uncommon. In 4 sections (2: NS, 2: LD) with marked T cell depletion, the "suppressor" subclass formed the predominant T cell population.

*Leu-7 positive cells* (Table V) Leu-7 positive lymphocytes were sparsely distributed in T and B cell predominant areas of 26/33 sections but were concentrated in residual germinal centres, where these were present. In the remaining 6 instances, Leu-7 positive cells were increased in number. In 2 cases of nodular LP, large numbers of Leu-7 positive cells were concentrated in the predominantly B lymphocyte nodules while in 1 case of NS (1 of 2 with intense T cell reactions) similar numbers of Leu-7 positive cells were observed in aggregates, near HD cells. In parts of each of these sections, their numbers accounted for over 30% of the lymphoid population and within those areas, Leu-7 positive cells frequently formed attachments with HD cells, resulting in partial or complete "rosettes" (Figure 4). In both of these cases it was impossible to exclude the possibility that a proportion of Leu-7 positive cells also expressed T cell antigens, since T cells within the same areas also formed "rosettes" with HD cells.

*Individual reactions of Hodgkin's cells*
(Tables VIA and B)

Reactions with anti HLA-DR, OKT9 and PNL were of moderate to marked intensity (Figures 5, 6 and 7 respectively). Condensation of cytoplasmic staining was particularly noticeable with both

**Figure 5** Positive staining of an HD cell (arrowed) with anti HLA-DR antibody, CA2-11 in a case of NS ( × 63 obj).

**Figure 6** Positive staining of HD cells with OKT9 in a case of LP ( × 63 obj).

**Figure 7** Staining of HD cells with PNL in a case of MC ( × 63 obj).

**Figure 8** Staining of an HD cell with B1 in a case of NS (cellular phase) × 63 obj. On other case of LD histology, also revealed positive staining of HD cells with B1. B1 positive cells are HD cells ( × 63 obj).

OKT9 and PNL. Diffuse staining of tissue histiocytes was noted with both OKT9 and anti HLA-DR but was generally weak. In contrast to the tissue histiocytes, however, HD cells did not react with anti monocyte/macrophage antibodies, OKM1 and Mo-2. However, these antibodies frequently resulted in moderately heavy staining of the intercellular matrix, particularly where this was accompanied by fibrosis.

These patterns of reactivity were typical of all morphological variants of the Sternberg–Reed cell and of all histological types. However, in 2 cases (1: NSCP, 1: LD), the majority of HD cells showed clear surface membrane reactions with the anti B lymphoid antibody, B1 (Figure 8). In neither of these 2 cases (which were morphologically and immunohistologically otherwise typical of HD) nor in any other instance was there detectable expression of surface immunoglobulins by HD cells.

*Effects of preceding therapy*

No clear effect of previous therapy on the phenotypic patterns found in Hodgkin's tissue was demonstrated. In one remarkable instance, a patient who had received radiotherapy for localised HD with LP histology relapsed after an interval of 25 years with advanced HD of LD histology.

### Discussion

The results of this study broadly confirm previous reports of the phenotype of HD cells and their relationships with T cells. In addition, several new

**Table V** Interactions of HD cells with Leu-7 positive lymphocytes

| *Group* | *no.* | *Adherence to HD cells* | *Distribution in tissue* |
|---|---|---|---|
| 1 | 26<br>(LP: 2)<br>(NS: 21)<br>(MC: 2)<br>(LD: 1) | nil | +/++ |
| 2 | 3<br>(NSCP: 1)<br>(NS: 1)<br>(LD: 1) | occasional | +++ |
| 3 | 4<br>(LP: 2)<br>(NS: 2) | frequent, with formation of partial or complete "rosettes" | ++++ |

\+ = scanty: 1% or less, all lymphoid cells.
++ = moderate: 1–5%.
+++ = increased: 5–10%.
++++ = marked increase, accounting for 10–30% all lymphoid cells.

**Table VIA** Individual positive reactions of HD cells

| *Reagent* | *no.* | *% Positive score per section* |
|---|---|---|
| CA2-11 | 28 (LP: 2)<br>(NS: 22)<br>(MC: 2)<br>(LD: 2) | 75–100 |
| | 2 (LP: 1)<br>(NS: 1) | 50–75 |
| | 2 (NS: 2) | <50 |
| | 1 (NS: poor section) | equivocal |
| OKT9 | 27 (LP: 4)<br>(NS: 19)<br>(MC: 2)<br>(LD: 2) | 75–100 |
| | 1 (NS) | 50 |
| | 5 (NS: 5) | equivocal |
| B1 | 2 (NSCP: 1)<br>(LD: 1) | 75–100 |
| | 1 (NS) | equivocal |
| | 30 | 0 |
| PNL | 27 (LP: 3)<br>(NS: 20)<br>(MC: 2)<br>(LD: 2) | 75–100 |
| | 3 (NS) | 50 |
| | 3 (NS: 2)<br>(LP: 1) | <50 |

**Table VIB** Negative reactions

| | |
|---|---|
| 1 | All T cell antibodies |
| 2 | OKT6 |
| 3 | Monocyte antibodies, OKM1 and Mo-2 |
| 4 | Immunoglobulin antibodies |
| 5 | J5 |

findings, taken together with other recently published work, provide further support for the thesis that the HD cell derives from a precursor normally resident in germinal follicles and indeed suggest that this may be a cell closely related to the B lymphoid lineage. The evidence for this hypothesis, as derived from the present study, include the following findings: (i) the constant association of HD cells with the mantle zones of secondary follicles; (ii) the detection of B cell antigens on HD cells with B1 in 2 cases of otherwise typical HD; (iii) the characteristic cytoplasmic staining of HD cells with peanut lectin. As argued below, the relationships of HD cells with T cells and, occasionally, Leu-7 positive cells provide additional although circumstantial evidence for this hypothesis.

The constant infiltration of residual secondary follicles by HD cells, as well as the finding of characteristic pseudo follicular B cell aggregates (also invariably infiltrated by HD cells), suggest a close involvement of the B cell compartment in the pathological process in HD. Preferential involvement of B cell areas by HD was reported by Cossman *et al.* (1977). Halie *et al.* (1978) also reported focal involvement of splenic B follicles by HD.

Considerable interest has been focussed on the role of germinal follicles in the pathogenesis of nodular LP ("nodular paragranuloma") (Lennert & Muller-Hermelink, 1975; Muller-Hermelink & Lennert, 1978). Indeed, the resemblance of nodular LP to "progressive germinal centre transformation" led to speculation (Lennert *et al.*, 1979; Poppema *et al.*, 1979) that the features were more suggestive of a B cell lymphoma than a true subtype of HD. The results of the present study, however, suggest that the immunohistological characteristics of nodular LP represent one part of the spectrum of interrelationships of HD cells and B follicles.

Reactions of HD cells with the B cell specific antibody B1 (Stashenko *et al.*, 1980) were first reported from this centre, in one case of NS HD in the "cellular phase" (Dorreen *et al.*, 1983), and now also in a case of LD. It is not clear why HD cells in so few of the cases in this study reacted with B1. However, Stuart *et al.* (1979, 1982) who previously reported positive reactions of HD cells with a heterologous B cell antiserum have since also described 4 cases in which positive reactions of HD cells with monoclonal B cell antibodies, B1, FMCl and FMC7 were observed (Stuart *et al.*, 1983). In accordance with the results of our larger study, no expression of surface Ig was detected on HD cells.

Peanut lectin was avidly bound by HD cells. Unlike a recent study (Bramwell *et al.*, 1982) which reported predominantly nuclear binding of PNL, the distribution of staining, in this study, was limited entirely to the cell membrane and/or cytoplasm. In reactive lymph nodes PNL is bound in germinal centres by B lymphoid and reticular cells bearing the terminal carbohydrate residue, galactose-N-acetyl glucosamine (Rose *et al.*, 1981).

Interactions of T cells and HD cells have been widely reported (Kadin *et al.*, 1974; Stuart *et al.*, 1977; Payne *et al.*, 1980; Poppema *et al.*, 1979 , 1982). Poppema *et al.* (1982) and Borowitz *et al.* (1982) first demonstrated that the interacting T cell population belonged to the "helper" subclass. The functional significance of these relationships is not clear although Stuart *et al.* (1977) suggested they might represent a disordered form of cellular immunological cooperation. Moreover, the T "helper" subpopulation appears to be a necessary component of B follicular development (Bhan *et al.*, 1981; Poppema *et al.*, 1980; Stein *et al.*, 1980).

The few instances in which Leu-7 positive cells formed similar clustering around HD cells, were intriguing findings. These observations have not previously been reported, although one study (Lusheng & Whiteside, 1983) described increased numbers of Leu-7 positive cells in HD tissue. Leu-7 (anti HNK-1) has been shown to define a population of human lymphoid cells with the functional attributes of "natural killer" (NK) and "killer" (K) cells (Abo & Balch, 1981). In normal lymphoid tissue, Leu-7 positive cells are predominantly localised in germinal centres (Banerjee & Thibert, 1983). Since large numbers of Leu-7 positive cells were observed in the predominantly B cell nodules of 2 cases of nodular LP these observations are of considerable interest. Because of the overall lack of clinical correlation, it is unlikely that these associations reflect important NK-mediated cytotoxicity although there is evidence (Vodinelich *et al.*, 1983) that "NK" cells interact with the transferrin receptor, ubiquitously expressed on HD cells.

The growth of a cell line derived from Sternberg–Reed cells (Diehl *et al.*, 1982) has led to the establishment of a monoclonal antibody (Schwab *et al.*, 1982) reportedly reactive with all HD cell variants (Stein *et al.*, 1982 ) and a small population of cells in the mantle zones of normal secondary follicles in lymphoid tissue (Stein *et al.*, 1982 ). The latter group of workers, however, also reported positive reactions of HD cells with monoclonal antibodies specific to cells of granulopoietic lineage. In common with these authors we did not observe positive reactions with monocyte antibodies, OKM1 and Mo-2. Reactions of HD cells with an anti dendritic reticulum cell antibody are also reportedly negative (Stein *et al.*, 1982).

Reactions of HD cells with anti HLA-DR have been extensively reported (Kadin & Billing, 1978; Poppema *et al.*, 1982; Borowitz *et al.*, 1982; Stuart *et al.*, 1982). This does not indicate lineage specificity, however, since HLA-DR may be expressed on B lymphocytes and reticular cells, (Poppema *et al.*, 1981) some monocytes (Yamashita *et al.*, 1977) and "activated" T cells (Evans *et al.*, 1978).

The transferrin receptor (recognised by the antibody, OKT9) is an antigen ubiquitously associated with cellular proliferation (Trowbridge & Omary, 1981; Sutherland *et al.*, 1981). Little information exists on the expression of this antigen by HD cells, although Borowitz *et al.* (1982), reported some positive reactions with an anti transferrin receptor antibody. In this study, the great majority of HD cells reacted positively with OKT9. These reactions may reflect the proliferative potential common to all variants of the Sternberg–Reed cell. However, HD cells are relatively few in number, compared with other components of the cellular reaction, in keeping with the relatively slow progression of disease. In aggressive and rapidly dividing lymphomas, on the other hand, cells reactive with OKT9 are present in raised numbers (Habeshaw *et al.*, 1983*a*, *b*).

The phenotypic expressions of Sternberg–Reed cells and morphological variants documented in this study, as well as their interactions with T and B lymphocytes suggest they are an antigenically homogeneous group of common derivation, although they do not clearly indicate their cellular lineage. The functional nature of the reactions of T "helper" cells and, occasionally, Leu-7 positive ("NK") cells with HD cells is not clear but suggests a cooperative reaction of immunological importance. The frequent occurrence of HD cells in B follicular structures, together with the finding that in 2 cases these were positive with the B lymphocyte antibody Bl, are intriguing findings and may point to a B lymphoid lineage, or derivation from a common B lineage precursor.

We are grateful to Mr W.S. Shand, consultant surgeon, under whose care all patients were admitted for lymph node biopsy and/or staging laparotomy.

### References

ABO, T. & BALCH, C.M. (1981). A differentiation antigen of human NK and K cells identified by a monoclonal antibody (HNK-1). *J. Immunol.*, **127**, 1024.

AGLIOZZO, C.M. & REINGOLD, I.M. (1971). Infectious mononucleosis simulating Hodgkin's disease: a patient with Reed–Sternberg cells. *Am. J. Clin. Pathol.*, **56**, 730.

ANAGNOSTOU, D., PARKER, J.W., TAYLOR, C.R., TINDLE, B.H. & LUKES, R.J. (1977). Lacunar cells of nodular sclerosing Hodgkin's disease. *Cancer*, **39**, 1032.

BANERJEE, B. & THIBERT, R.F. (1983). Natural killer-like cells found in B-cell compartments of human lymphoid tissues. *Nature*, **304**, 270.

BEVERLEY, P.C.L. & CALLARD, R.E. (1981). Distinctive functional characteristics of human "T" lymphocytes defined by E rosetting or a monoclonal anti-T cell antibody. *Eur. J. Immunol.*, **11**, 329.

BHAN, A.K., NADLER, L.M., STASHENKO, P., McCLUSKEY, R.T. & SCHLOSSMAN, S.F. (1981). Stages of B cell differentiation in human lymphoid tissue. *J. Exp. Med.*, **154**, 737.

BINIAMINOV, M. & RAMOT, B. (1974). Possible T lymphocyte origin of Reed–Sternberg cells. *Lancet*, **i**, 368.

BOROWITZ, M.J., CROKER, B.P. & METZGAR, R.S. (1982). Immunohistochemical analysis of the distribution of lymphocyte subpopulations in Hodgkin's disease. *Cancer Treat. Rep.*, **66**, 667.

BRAMWELL, V.H.C., CROWTHER, D., GALLAGHER, J. & STODDART, R.W. (1982). Studies of lectin binding to normal and neoplastic lymphoid tissues. I. Normal nodes and Hodgkin's disease. *Br. J. Cancer*, **46**, 568.

BROOKS, J.S.J. (1979). Leukophagocytosis by Sternberg–Reed cells in Hodgkin's disease. *N. Engl. J. Med.*, **300**, 1115.

CARBONE, P.P., KAPLAN, H.S., MUSSHOFF, K., SMITHERS, D.W. & TUBIANA, M. (1971). Report of the committee on Hodgkin's disease staging. *Cancer Res.*, **31**, 1860.

CHARRON, D.J. & McDEVITT, H.O. (1979). Analysis of HLA-D region – associated molecules with monoclonal antibody. *Proc. Natl Acad. Sci.*, **76**, 6567.

COSSMAN, J., DEEGAN, M.J. & SCHNITZER, B. (1977). Complement receptor B lymphocytes in nodular sclerosing Hodgkin's disease. *Cancer*, **39**, 2166.

CURRAN, R.C. & JONES, E.L. (1977). Dendritic cells and B lymphocytes in Hodgkin's disease. *Lancet*, **2**, 349.

CURRAN, R.C. & JONES, E.L. (1978). Hodgkin's disease: an immunohistochemical and histological study. *J. Pathol.*, **125**, 39.

DE VITA, V.T. (1973). Lymphocyte reactivity in Hodgkin's disease: a lymphocyte civil war. *N. Engl. J. Med.*, **289**, 801.

DIEHL, V., KIRCHNER, H.H., BURRICHTER, H., STEIN, H., FONATSCH, C., GERDES, J., SCHAADT, M., HEIT, W., UCHANSKA-ZIEGLER, B., ZIEGLER, A., HEINTZ, F. & SUENO, K. (1982). Characteristics of Hodgkin's disease-derived cell lines. *Cancer Treat. Rep.*, **66**, 615.

DORREEN, M.S., HABESHAW, J.A., STANSFELD, A.G. & LISTER, T.A. (1983). Characteristics of Sternberg–Reed cells: an immunohistological study of Hodgkin's disease. *Proc. Am. Assoc. Cancer Res.*, **24**, 820.

EVANS, R.L., FALDETTA, T.J., HUMPHREYS, R.E., PRATT, D.M., YUNIS, E.J. & SCHLOSSMAN, S.F. (1978). Peripheral human T cells sensitised in mixed leukocyte cultures synthesise and express Ia-like antigens. *J. Exp. Med.*, **148**, 1440.

GARVIN, A.J., SPICER, S.S., PARMLEY, R.T. & MUNSTER, A.M. (1974). Immunohistochemical demonstration of IgG in Reed–Sternberg and other cells in Hodgkin's disease. *J. Exp. Med.*, **139**, 1077.

GREAVES, M.F., DELIA, D., ROBINSON, J., SUTHERLAND, R. & NEWMAN, R. (1981). Exploitation of monoclonal antibodies: a "who's who" of haematopoietic malignancy. *Blood Cells*, **7**, 257.

HABESHAW, J.A., BAILEY, D., STANSFELD, A.G. & GREAVES, M.F. (1983*a*). The cellular content of non Hodgkin lymphomas: a comprehensive analysis using monoclonal antibodies and other surface marker techniques. *Br. J. Cancer*, **47**, 327.

HABESHAW, J.A., LISTER, T.A., STANSFELD, A.G. & GREAVES, M.F. (1983*b*). Correlation of transferrin receptor expression with histological class and outcome in non-Hodgkin lymphoma. *Lancet*, **i**, 498.

HALIE, M.R., THIADEUS, J., EIBERGEN, R. & VAN DEN BROCK, A.A. (1978). Hodgkin's disease in the spleen. Investigation of Hodgkin's foci and areas for the immune response. *Virchows Arch. B. Cell Pathol.*, **27**, 39.

HARTSOCK, R.J. (1968). Postvaccinial lymphadenitis. Hyperplasia of lymphoid tissue that simulates malignant lymphomas. *Cancer*, **21**, 632.

KADIN, M., NEWCOM, S.R., GOLDA, S.R. & STITES, D.P. (1974). Origin of Hodgkin's cell. *Lancet*, **ii**, 167.

KADIN, M.E., STITES, D.P., LEVY, R. & WARNKE, R. (1978). Exogenous immunoglobulin and the macrophage origin of Reed–Sternberg cells in Hodgkin's disease. *N. Engl. J. Med.*, **299**, 1208.

KADIN, M.E. & BILLING, R.J. (1978). B lymphocyte antigens in the differential diagnosis of human neoplasia. *Blood*, **51**, 813.

KADIN, M.E. (1982). Possible origin of the Reed–Sternberg cell from an interdigtating reticulum cell. *Cancer Treat. Rep.*, **66**, 601.

KAPLAN, H.S. (1981). In: *Hodgkin's Disease* (second edition) Harvard, University Press.

KATZ, D.R. (1981). The macrophage in Hodgkin's disease. *J. Pathol.*, **133**, 145.

LEECH, J. (1973). Immunoglobulin-positive Reed–Sternberg cells in Hodgkin's disease. *Lancet*, **i**, 265.

LENNERT, K. & MULLER-HERMELINK, H.K. (1975). Lymphocyten und ihre funktionsformen – morphologie, organisation und immunologische bedeutung (lecture). *Verhandlungen der Anatomischen Gesellschaft*, **69**, 19.

LENNERT, K., KAISERLING, E. & MULLER-HERMELINK, H.K. (1979). Malignant lymphomas: models of differentiation and cooperation of lymphoreticular cell. Cold Spring Harbour conferences on cell proliferation. Vol. 5. Book B. (Eds. Clarkston *et al.*).

LUSHENG, S. & WHITESIDE, T.L. (1983). Tissue distribution of human NK cells studied with anti Leu-7 monoclonal antibody. *J. Immunol.*, **130**, 2149.

LUKES, R.J., BUTLER, J.J. & HICKS, E.B. (1966). Natural history of Hodgkin's disease as related to its pathologic picture. *Cancer*, **19**, 317.

LUKES, R.J., CRAVER, L.F., HALL, T.C., RAPPAPORT, H. & RUBIN, P. (1966). Report of the nomenclature committee. *Cancer Res.*, **26**, 1311.

LUKES, R.J., TINDLE, B.H. & PARKER, J.W. (1969). Reed–Sternberg-like cells in infectious monocucleosis. *Lancet*, **ii**, 1003.

MULLER-HERMELINK, H.K. & LENNERT, K. (1978). The cytologic, histologic and functional bases for a modern classification of lymphomas. In: *Malignant Lymphomas Other than Hodgkin's Disease*. (Eds. Lennert *et al.*), p. 1 Springer, New York.

ORDER, S.E. & HELLMAN, S. (1972). Pathogenesis of Hodgkin's disease. *Lancet*, **i**, 571.

PAYNE, S.V., JONES, D.B., HAEGERT, D.G. & WRIGHT, D.H. (1976). T and B lymphocytes and Reed–Sternberg cells in Hodgkin's disease lymph nodes and spleens. *Clin. Exp. Immunol.*, **24**, 280.

PAYNE, S.V., NEWELL, D.G., JONES, D.B. & WRIGHT, D.H. (1980). The Reed–Sternberg cell/lymphocyte interaction. *Am. J. Pathol.*, **100**, 7.

PAYNE, S.V., WRIGHT, D.H., JONES, K. & JUDD, M.A. (1982). Macrophage origin of Reed–Sternberg cells: an immunohistochemical study. *J. Clin. Pathol.*, **35**, 159.

POPPEMA, S., ELEMA, J.D. & HALIE, M.R. (1978). The significance of intracytoplasmic proteins in Reed–Sternberg cells. *Cancer*, **42**, 1793.

POPPEMA, S., ELEMA, J.D. & HALIE, M.R. (1979*a*). The localisation of Hodgkin's disease in lymph nodes. A study with immunohistological, enzyme histochemical and rosetting techniques on frozen sections. *Int. J. Cancer*, **24**, 532.

POPPEMA, S., KAISERLING, E. & LENNERT, K. (1979*b*). Hodgkin's disease with lymphocytic predominance, nodular type (nodular paragranuloma) and progressively transformed germinal centres – a cytohistological study. *Histopathology*, **3**, 295.

POPPEMA, S., KAISERLING, E. & LENNERT, K. (1979*c*). Nodular paragranuloma and progressively transformed germinal centres. Ultrastructural and immunohistologic findings. *Virchow's Arch. B. Cell. Pathol.*, **31**, 211.

POPPEMA, S., BHAN, A.K., REINHERZ, E.L., McCLUSKEY, R.T. & SCHLOSSMAN, S.F. (1981). Distribution of T cell subsets in human lymph nodes. *J. Exp. Med.*, **153**, 30.

POPPEMA, S., BHAN, A.K., REINHERZ, E.L., POSNER, M.R. & SCHLOSSMAN, S.F. (1982). *In situ* characterisation of cellular constituents in lymph nodes and spleens involved by Hodgkin's disease. *Blood*, **59**, 226.

RESNICK, G.D. & NACHMAN, R.L. (1981). Reed–Sternberg cells in Hodgkin's disease contain fibronectin. *Blood*, **57**, 339.

RODER, J.C. & PROSS, H.F. (1982). The biology of the human natural killer cell. *J. Clin. Immunol.*, **2**, 249.

ROSE, M.L., HABESHAW, J.A., KENNEDY, R., SLOANE, J., WILTSHAW, E. & DAVIES, A.J.S. (1981). Binding of peanut lectin to germinal-centre cells: a marker for B-cell subsets of follicular lymphoma? *Br. J. Cancer*, **44**, 68.

SALTZSTEIN, S.L., JAUDON, J.C., LUSE, S.A. & ACKERMAN, L.V. (1958). Lymphadenopathy induced by ethotoin (Peganone). Clinical and pathological mimicking of malignant lymphoma. *J.A.M.A.*, **167**, 1618.

SALTZSTEIN, S.L. & ACKERMAN, L.V. (1959). Lymphadenopathy induced by anti-convulsant drugs and mimicking clinically and pathologically malignant lymphomas. *Cancer*, **12**, 164.

SCHWAB, U., STEIN, H., GERDES, J., LEMKE, H., KIRCHNER, H., SCHAADT, M. & DIEHL, V. (1982). Production of a monoclonal antibody specific for Hodgkin and Sternberg–Reed cells of Hodgkin's disease and a subset of normal lymphoid cells. *Nature*, **299**, 65.

STASHENKO, P., NADLER, L.M., HARDY, R. & SCHLOSSMAN, S.F. (1980). Characterisation of a human B lymphocyte specific antigen. *J. Immunol.*, **125**, 1678.

STEIN, H., PAPADIMITRIOU, C.S., BOUMAN, H., LENNERT, K. & FUCHS, J. (1978). Demonstration of immunoglobulin production by tumour cells in non-Hodgkin's and Hodgkin's malignant lymphomas and its significance for their classification. *Recent Results Cancer Res.*, **1**, 158.

STEIN, H., BONK, A., TOLKSDORF, G., LENNERT, K., RODT, H. & GERDES, J. (1980). Immunohistologic analysis of the organisation of normal lymphoid tissue and non-Hodgkin's lymphomas. *J. Histochem. Cytochem.*, **26**, 746.

STEIN, H., GERDES, J., KIRCHNER, H., DIEHL, V., SCHAADT, M., BONK, A. & STEFFEN, T. (1981). Immunohistological analysis of Hodgkin's and Sternberg–Reed cells: detection of a new antigen and evidence of selective IgG uptake in the absence of B cell, T cell and histiocytic markers. *J. Cancer Res. Clin. Oncol.*, **101**, 125.

STEIN, H., UCHANSKA-ZIEGLER, B., GERDES, J., ZIEGLER, A. & WERNET, P. (1982*a*). Hodgkin and Sternberg–Reed cells contain antigens specific to late cells of granulopoiesis. *Int. J. Cancer*, **29**, 283.

STEIN, H., GERDES, J., SCHWAB, U., LEMKE, H., MASON, D.Y., ZIEGLER, A., SCHIENLE, W. & DIEHL, V. (1982*b*). Identification of Hodgkin and Sternberg–Reed cells as a unique cell type derived from a newly detected small-cell population. *Int. J. Cancer*, **30**, 445.

STUART, A.E., WILLIAMS, A.R.W. & HABESHAW, J.A. (1977). Rosetting and other reactions of the Reed–Sternberg cell. *J. Pathol.*, **122**, 81.

STUART, A.E. & DEWAR, A.E. (1979). Properties of anti-hairy cell serum. *Br. J. Haematol.*, **41**, 163.

STUART, A.E., JACKSON, E. & MORRIS, C.S. (1982). The reaction of xenogenic and monoclonal antisera with Reed–Sternberg cells. *J. Pathol.*, **137**, 129.

STUART, A.E., VOLSEN, S.G. & ZOLA, H. (1983). The reactivity of Reed–Sternberg cells with monoclonal antisera at thin section and ultrastructural levels. *J. Pathol.*, **141**, 71.

STRUM, S.B., PARK, J.K. & RAPPAPORT, H. (1970). Observations of cells resembling Sternberg–Reed cells in conditions other than Hodgkin's disease. *Cancer*, **26**, 176.

SU-MING, H., RAINE, M.S. & FANGER, H. (1981. The use of antiavidin antibody and avidin-biotin-peroxidase complex in immunoperoxidase technics. *Am. J. Clin. Pathol.*, **75**, 816.

SUTHERLAND, R., DELIA, D., SCHNEIDER, C., NEWMAN, R., KEMSHEAD, J. & GREAVES, M. (1981). Ubiquitous cell-surface glycoprotein on tumour cells is proliferation associated receptor for transferrin. *Proc. Natl Acad. Sci.*, **78**, 4515.

TAYLOR, C.R. (1974). The nature of Reed–Sternberg cells and other malignant reticulum cells. *Lancet*, **ii**, 802.

TAYLOR, C.R. (1976). An immunohistological study of follicular lymphoma, reticular cell sarcoma and Hodgkin's disease. *Eur. J. Cancer*, **12**, 61.

TAYLOR, C.R. (1978). Upon the nature of Hodgkin's disease and the Reed–Sternberg cell. *Recent Results Cancer Res.*, **1**, 214.

TINDLE, B.H., PARKER, J.W. & LUKES, R.J. (1972). "Reed–Sternberg cells" in infectious mononucleosis? *Am. J. Clin. Pathol.*, **58**, 607.

TROWBRIDGE, I.S. & OMARY, M.B. (1981). Human cell surface glycoprotein related to cell proliferation is the receptor for transferrin. *Proc. Natl Acad. Sci.*, **78**, 3039.

VERBI, W., GREAVES, M.F., SCHNEIDER, C., KOUBEK, K., JANOSSY, G., STEIN, H., KUNG, P.C. & GOLDSTEIN, G. (1982). Monoclonal antibodies OKT11 and OKT11a have pan-T reactivity and block sheep erythrocyte receptors. *Eur. J. Immunol.*, **12**, 81.

VODINELICH, L., SUTHERLAND, R., SCHNEIDER, C., NEWMAN, R. & GREAVES, M. (1983). Receptor for transferrin may be a "target" structure for natural killer cells. *Proc. Natl Acad. Sci.*, **80**, 835.

YAMASHITA, U. & SHEVACH, E. (1977). The expression of Ia antigens on immunocompetent cells in the guinea pig. II. Ia antigens on macrophages. *J. Immunol.*, **119**, 1584.